# Idiopathic toe walkers: Do they need surgery?

**DOI:** 10.1371/journal.pone.0331099

**Published:** 2025-09-30

**Authors:** Alice Bonnefoy-Mazure, Marys Revaz, Camille Leroquais, Geraldo de Coulon, Pierre Lascombes, Stéphane Armand

**Affiliations:** 1 Laboratory of Kinesiology, Geneva University Hospitals and University of Geneva, Geneva, Switzerland; 2 Research Center of skeletal Muscle and Movement, Geneva University and University of Geneva Hospitals, Geneva, Switzerland; 3 Pediatric Orthopedic Service, Department of Child and Teenage Medicine, Geneva University Hospitals and University of Geneva, Geneva, Switzerland; 4 Geneva University Hospitals and University of Geneva, Geneva, Switzerland; 5 Honorary Professor of Paediatric Orthopaedics of the University of Medicine, Nancy, France; Aichi Prefectural Mikawa Aoitori Medical and Rehabilitation Center for Developmental Disabilities, JAPAN

## Abstract

**Aim:**

The aims of study were: to observe gait parameters evolution for group of patients with surgery and conservative treatment; to compare these parameters for a group of matched patients with surgery and without; to compare these parameters with a group of asymptomatic children.

**Background:**

Depending of the importance of the equinus in idiopathic toe walking (ITW) patients, conservative or surgical treatment could be proposed. Currently, there is no consensus about the treatment of ITW.

**Methods:**

ITW patients with surgical or conservative treatment and with two clinical gait analysis (CGA before and after treatment) were selected. Gait parameters have been used as: presence of first ankle rocker, peak ankle power generation, ankle passive dorsiflexion with extended and flexed knee. Paired and Unpaired t-test were used to analyse differences between the 2 CGA, between groups of treatment and with asymptomatic children.

**Results:**

Sixteen ITW patients were treated surgically with a mean age at baseline of 8.6y and a follow-up time of 2.1y. Thirteen ITW patients were treated conservatively with mean age at baseline of 6.5y and a follow-up time of 3.0y. Only surgery group had a significant improvement in passive ankle dorsiflexion. Both groups significantly improved their parameters. Ten patients in each group were similar in terms of passive ankle dorsiflexion at the first CGA. Passive dorsiflexion and ankle power were significantly greater for the surgery group at the second CGA. Parameters were significantly lower compared to asymptomatic children.

**Conclusions:**

Regardless of the treatment, the gait quality of the ITW patients improves over time but does not recover the gait of healthy children.

**Level of evidence:**

This study was a therapeutic retrospective comparative study (level III)

## Introduction

Idiopathic toe walking (ITW) is a diagnostic of exclusion when toe walking persists after the age of 5 years in the absence of any neurological or orthopedic abnormalities [1–3]. Ankle equinus is one of the main characteristics of this population. Depending on the importance of this ankle equinus, lower limb or foot pain can be present with also poor motor skills [4,5]. In addition, ITW can have negative consequences on child’s gait and skeletal development, in particular on the talus [1,6]. Currently, there is no consensus about the treatment of ITW [2]. Non-operative treatments include reassurance alone, based on the assumption that ITW often improves without any intervention, conservative treatments by physiotherapy, orthosis and/or cast and pharmacological treatment by injection of Botulinum Toxin A [2,7,8]. Surgical treatment by Achilles tendon lengthening is usually preferred when there is a severe equinus contracture with a failure of the other treatments [3,9].

Based on instrumented clinical gait analysis (CGA), few studies showed beneficial post-surgical treatment results [9–12]. Surgical treatment seems to allow better passive ankle dorsiflexion and parental satisfaction than conservative treatment but the number and the quality of studies investigating ITW treatments are limited and there is still no consensus about the choice of these treatments [3,5,13].

Thus, the primary objectives of this study were to: a) assess gait modifications in each group of treatment, after a surgery and after conservative treatment in ITW and b) compare gait modifications in function of the treatment (surgical vs. conservative) in ITW patients with similar passive ankle dorsiflexion at baseline (first CGA). The secondary objective was to assess gait recovery in ITW with surgery and with conservative treatment compared to asymptomatic children.

## Materials and methods

### Design

This retrospective study reviewed all patients with a diagnostic of ITW and having two gait analysis examination between 01.01.2006 and 31.12.2022. All the retrospective data has been accessed in 2023. All procedures performed in studies involving human participants were in accordance with the ethical standards of the institutional and/or national research committee and with the 1964 Helsinki declaration and its later amendments or comparable ethical standards. The study has been approved by the Cantonal Commission on Ethics in Human Research in Geneva (CER no. 2018−00229). Informed consent was obtained from all participants and their respective legal guardians since the approval (March 2018). For instrumented gait analysis performed prior to this date, a consent exemption was granted by the local ethics committee.

### Participants

Diagnostic of ITW was defined as a bilateral absence of the first rocker and/or a premature elevation of the heel after the age of 5 years, in the absence of any neurological or orthopedic abnormalities^1–3^. Inclusion criteria were a diagnostic of ITW by a pediatric orthopedic surgeon, with surgical or conservative treatment, and with, two CGA sessions (before and after treatment).

Exclusion criteria were an age inferior to 6 years or superior to 14 years at the first gait analysis, and more than 4 years between the two CGA sessions.

The gait data of 21 asymptomatic children were used as kinematic reference data in the gait laboratory and similar for age to the ITW patients post-treatment were used for comparison. Among them, 16 asymptomatic children were used as kinetic reference data.

### Treatments

Pediatric orthopedic surgeons proposed a surgical treatment looking at four criteria: 1) retraction of triceps surae (meaning a passive ankle dorsiflexion inferior to 0° at one side when knee are extended), 2) presence of pain or instability during the gait, 3) absence or limitation of the first rocker and 4) impossibility to walk with a normal gait pattern at the request of the clinician – “best walk” (reduced maximal dorsiflexion during walking, pejoration of the global gait or important decrease in the walking speed). Surgical treatment was a percutaneous Achilles tendon lengthening by Hoke technique [14]. One patient was operated under Vulpius technique, he was excluded from the study [15].

Pediatric orthopedic surgeons proposed a conservative treatment for ITWs that do not fall within the surgical criteria. Conservative treatments consisted of physiotherapy and casts.

### Gait evaluation

The patients were asked to walk barefoot on a 10-m walkway at their self-selected speed. Markers were placed on lower limbs and pelvis in accordance with the Davis protocol, and their trajectories were recorded at 100 Hz by a 12-camera motion measurement system (Vicon Mx3 + , Oxford Metrics, UK, until 2015 and Qualisys Oqus7 + , Qualisys, Sweden, after 2015) [16]. Two force plates (AMTI-AccuGait, AMTI, USA) were used to record ground reaction forces at 1000 Hz. Visual 3D software (C-Motion, USA) was used to compute the kinematics and kinetics data according to the Conventional Gait Model [17] for at least 3 gait cycles by session. Data were time-normalized over one gait cycle. To evaluate the capacity of the patients to correct their gait pattern, walking trials have been recorded with the recommendation to do their “best walk” by striking the ground with the heel. Ankle kinematic and kinetic curves in the sagittal plane have been reported to reflect respectivelly the ankle plantar-dorsiflexion movements and moments during the gait cycle for ITW patients and asymptomatic children.

### Gait variable selection

Variable selection mainly stood on the Alvarez classification [18], based on the presence of first ankle rocker, the presence of early third ankle rocker and a predominant first ankle moment. Seven variables were chosen to assess the severity of ITW: the presence of first ankle rocker, the value and the time of maximal dorsiflexion during stance which reflect the third ankle rocker, the ratio between the two ankle maximal moments in the first and in the second half of the gait cycle, the peak ankle power generation, Ankle Gait Variable Score (GVS) and the modified GPS (mGPS without hip rotation) [10,11,18–20]. The reference dataset from Schwartz et al. (including 83 subjects) was used to compute mGPS scores [21]. The presence of the first ankle rocker was defined as a negative slope of the sagittal ankle kinematics during the first nine percent of the gait cycle, calculated as S = ΔA/Δt, with A the ankle kinematics and t the time [22].

Each variable was averaged for all gait cycles preoperatively and postoperatively for each patient. All the computations were performed with Matlab 2022a (MathWorks, USA) and the open-source Biomechanical ToolKit package for MATLAB [23].

### Clinical variable selection

At each CGA, the ankle passive dorsiflexion with extended and flexed knee were measured by physiotherapists with manual goniometer [24,25]. Age, sex, weight, and height were also included to characterize the cohort of the patients included in this study. Additionally, at the end of the visit, patients were asked: “On a scale from 0 (no pain) to 10 (worse pain), how would you rate your pain today during gait analysis?”. Responses were collected using a 10-point visual analogue scale (VAS), allowing us to assess the gait-related pain level.

### Data analysis

Each limb was considered independently. Variables cited above were compared in different groups in relation to the two major objectives:

a. In order to assess gait modifications in each group of treatment (surgical or conservative), preoperative data were compared with postoperative data, using a paired t-test.
b. In order to assess potential differences in the gait modifications for ITW patients depending on the treatment (surgical or conservative), a comparison of the data was done only for the patients matched on the mean ankle passive dorsiflexion (knee extended) at baseline and then the same postoperative data were compared, using an unpaired t-test.
c. In order to assess gait recovery in ITW, post-operative data were compared with asymptomatic children for each group of treatment children using an unpaired t-test.

Statistical analyses were performed using STATA software, version 13.1 (StataCorp LP, College Station, Texas, USA). Statistical significance was set at **p* *< 0.05 (two-sided).

## Results

**Fig 1:**
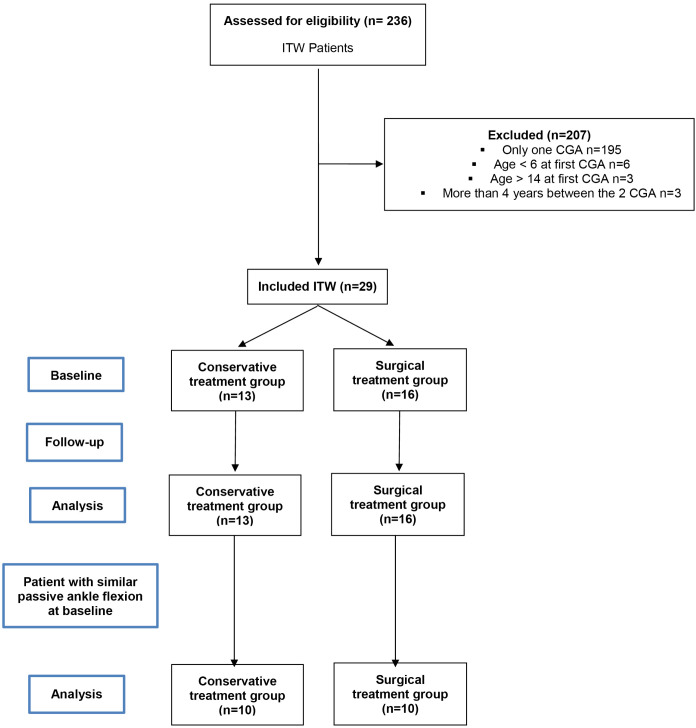
Flow chart of patient selection.

Based on the inclusion and exclusion criteria 29 ITW patients with 2 CGA (before and after treatments) were included (Fig 1). Sixteen patients were treated surgically (5 girls and 11 boys) with a mean age at baseline of 8.6 (1.9) years and a follow-up time of 2.1 (1.8) years. Thirteen patients were treated conservatively (5 girls and 8 boys) with mean age at baseline of 6.5 (1.9) years and a follow-up time of 3.0 (1.6) years. Twenty-one asymptomatic children with a mean age of 11.0 (1.8) years were selected in our “normal database” to be similar for age with ITW patients at the second CGA (Table 1).

**Table 1:** Idiopathic Toe-Walker (ITW) and healthy control participant’s characteristics.

	ITW patients with surgical treatment n = 16	ITW patients with conservative treatment n = 13	Asymptomatic children n = 21
**Age (years)**			
Baseline Visit	8.6 (1.9)	6.5 (0.4)	–
Surgery	9.3 (1.9)	–	–
Follow-up visit	10.7 (2.2)	9.5 (2.2)	11.0 (1.8)
Period between evaluations	2.1 (1.8)	3.0 (1.5)	–
**Gender (n)**			
Female	5	5	10
Male	11	8	11
**Weight (kg)**			
Baseline visit	31.7 (7.3)	29.7 (9.3)	–
Follow-up visit	40.9 (9.6)	45.6 (17.9)	39.0 (11.1)
**Height (m)**			
Baseline visit	1.31 (0.8)	1.3 (1.1)	–
Follow-up visit	1.46 (0.89)	1.46 (1.18)	1.49 (0.1)
Normalised walking speed*			
Baseline visit	0.46 (0.05)	0.48 (0.07)	–
Follow-up visit	0.46 (0.06)	0.45 (0.06)	0.41 (0.06)

*Data are given as: mean (standard deviation).*

*Normalised walking speed = $\frac{v}{\sqrt{g.l}}$ *with v the walking speed (m/s), l the leg length (m) and g = 9.81m/s*^*2*^*.*

### Gait modifications after surgical treatment

**Fig 2:**
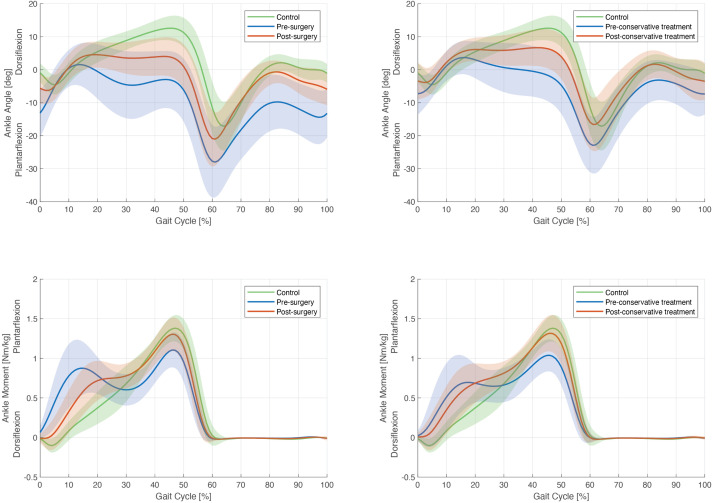
Graphical illustration of sagittal ankle angle and sagittal ankle moments in the surgical group (first column) and in the conservative treatment group (second column). The blue curves represent the data before surgery and conservative treatment and the red curves represent the same data after surgery and conservative treatement. The gray band represents sagittal ankle angle or moments based on normative data from the healthy group within two standard deviations of the mean.

Significant differences were found in surgical group pre- and post-surgery for all variables, except for the maximal ankle power (Table 2). Mean of ankle passive dorsiflexion improved, from 0.5° (10.8) to 10.3° (5.1) with extended knee and from 8° (8.5) to 16.9° (5.8) with flexed knee. mGPS decreased significantly (7.8° (1.8) to 6.1° (1.2)) showing a global improvement of gait quality after surgery with more specifically an ankle GVS that also decreased from 11.8° (5.8) to 6.7° (2.4), meaning an improvement of the ankle pattern of the gait^16^. The mean value of the slope of the sagittal ankle kinematics during the first nine percent of the gait cycle, representative of the first ankle rocker, became negative after the operation, passing from 0.4°/s (0.6) to −0.6°/s (0.7) indicating an increased presence of first ankle rocker. Maximal ankle dorsiflexion during stance improved from 4.6° (5.5) to 8.0° (4.1) as well as the time at maximal ankle dorsiflexion, improving from 21.4% (10) of the gait cycle to 30.8% (9.5), which indicated a later third rocker. Finally, the first/second moment ratio diminished after the operation, passing from 0.9 (0.2) to 0.6 (0.1) showing a less predominant first moment and thus an improvement of the dynamic function of the ankle-foot complex. Kinematic and kinetic curves showed an improvement post-surgery including: a better first rocker, a higher dorsiflexion during stance and swing phases and a reduced “double-bump” pattern for the ankle flexion moment. Finally, a reduction of the variability in these curves was also observed. (Fig 2-first column).

**Table 2:** Mean and SD of kinematic/kinetic and physical examination outcome measurements in ITW surgical (before and after the surgery of Achilles tendon lengthening) and in conservative group at the first and last Clinical Gait Analysis (CGA).

	Surgical treatment			Conservative treatment		
	First CGA n (legs) = 32		Last CGA n (legs) = 32	*p-values*	First CGA n (legs) = 26	Last CGA n (legs) = 26	*p-values*
Ankle passive dorsiflexion Extended knee (°)	**0.5 (10.8)**		**10.3 (5.1)**	*<0.05*	7.9 (6.9)	7.9 (6.9)	*1.00*
Ankle passive dorsiflexion Flexed knee (°)	**8.0 (8.5)**		**16.9 (5.8)**	*<0.05*	17.1 (8.5)	15.9 (7.1)	*0.56*
Ankle GVS (°)	**11.8 (5.8)**		**6.7 (2.4)**	*<0.05*	**9.7 (5.2)**	**6.3 (2.1)**	*<0.05*
GPS modified (°)	**7.8 (1.8)**		**6.1 (1.2)**	*<0.05*	**7.3 (1.6)**	**6.0 (1.4)**	*<0.05*
Maximal ankle dorsiflexion during stance phase of the gait (°)	**4.6 (5.5)**		**8.0 (4.1)**	*<0.05*	**5.4 (5.0)**	**8.7 (3.5)**	*<0.05*
Time at maximal ankle dorsiflexion during stance (% gait cycle)	**21.4 (10.0)**		**30.8 (9.5)**	*<0.05*	**25.2 (10.2)**	**32.9 (9.9)**	*<0.05*
Slope of the curve to estimate first rocker* (°/s)	**0.4 (0.6)**		**−0.6 (0.7)**	*<0.05*	**−0.2 (0.6)**	**−0.6 (0.7)**	*0.02*
First/second ankle moment ratio	**0.9 (0.2)**		**0.6 (0.1)**	*<0.05*	**0.8 (0.3)**	**0.6 (0.2)**	*0.01*
Maximal ankle power (W/kg)	3.3 (0.7)		3.7 (1.1)	*0.75*	3.2 (1.0)	3.0 (0.8)	*0.80*

*Slope of the sagittal ankle kinematics during the first nine percent of the gait cycle. A negative value indicates a plantarflexion movement and so the presence of the first ankle rocker.

### Gait modifications after conservative treatment

As for the group with surgical treatment, significant improvements were observed for all kinematic and kinetic outcomes (except for the maximal ankle power) showing also a global improvement of the gait quality for ITW with conservative treatment (Table 2). However, no significant improvements were found for the physical examination outcomes with similar values over time in terms of ankle passive movements (first CGA: 7.9° (6.9) vs. last CGA: 7.9° (6.9) with knee extended and first CGA: 17.1° (8.5) vs. last CGA: 15.9° (7.1) with knee flexed). As for surgical group, the Fig 2-second column, showed an improvement in the kinematic and kinetic curves and an improvement of the gait variability (Fig 2, second column).

### Comparison between surgical and conservative treatment

**Fig 3:**
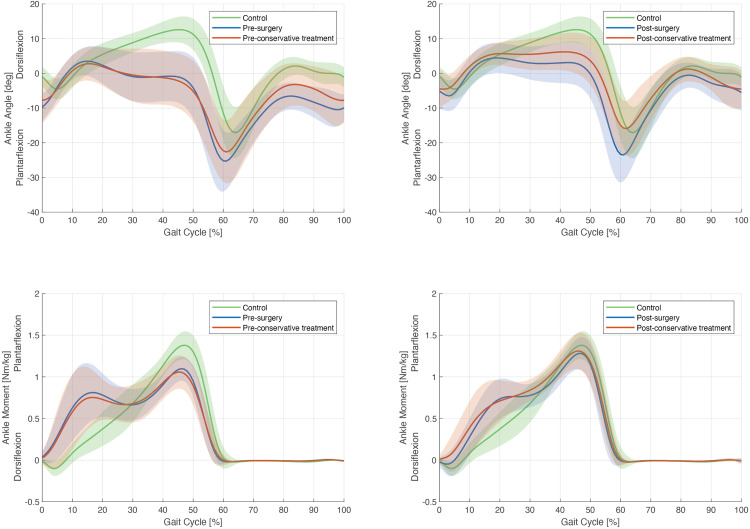
Graphical illustration of sagittal ankle angle and sagittal ankle moments (included only patients similar for the ankle passive dorsiflexion extended knee at baseline), in surgical and conservative treatments groups, in pre- (first column) and post-treatment (second column). The blue curves represent the data before surgery and conservative treatment and the red curves represent the same data after surgery and conservative treatement. The gray band represents sagittal ankle angle or moments based on normative data from the healthy group within two standard deviations of the mean.

For this comparison, 10 patients (20 legs) with matched ankle passive dorsiflexion with extended knee at baseline were selected in each group of treatment. Three girls and 7 boys were included in each group with younger patients for the conservative treatment at baseline (Table 3). In the ITW surgical group before treatment, 40% of patients reported pain with the majority of them (90%) that were not able to correct their gait (“best gait”). In the contrary, in the ITW conservative group, 20% of patients reported pain and the majority of them (70%) were able to correct their gait. It appears also that the ITW surgical group before treatment had a significantly lower ankle passive dorsiflexion with flexed knee compared to the group with conservative treatment (11.8° (6.5) vs. 16.0° (7.4)) and a first rocker significantly higher (0.4°/s (0.6) vs. −0.2°/s (0.6)). Kinematic and kinetic curves were similar at baseline (Fig 3 – first column).

**Table 3:** Mean and SD of kinematic/kinetic and physical examination outcome measurements in ITW surgical and in conservative groups similar for the ankle passive dorsiflexion extended knee at first and last Clinical Gait Analysis (CGA).

	First CGA			Last CGA		
	Surgical treatment n (legs) = 20	Conservative treatment n (legs) = 20	*p-values*	Surgical treatment n (legs) = 20	Conservative treatment n (legs) = 20	*p-values*
Age (years)	8.7 (2.1)	6.7 (2.1)	*<0.05*	10.9 (2.4)	9.9 (2.4)	*0.18*
Pain (%)	40%	20%	*–*	0%	20%	*–*
Ability to correct their gait (%)	10%	70%	*–*	100%	100%	*–*
Ankle passive dorsiflexion Extended knee (°)	5.5 (4.8)	6.5 (5.1)	*0.53*	**11.0 (4.8)**	**5.5 (5.6)**	*<0.05*
Ankle passive dorsiflexion Flexed knee (°)	**11.8 (6.5)**	**16.0 (7.4)**	*0.03*	**17.8 (5.7)**	**13.5 (5.6)**	*0.02*
Ankle GVS (°)	10.2 (5.6)	9.9 (3.7)	*0.83*	7.0 (2.5)	6.3 (2.2)	*0.35*
GPS modified (°)	7.4 (1.6)	7.1 (1.5)	*0.59*	6.2 (1.3)	5.8 (1.3)	*0.36*
Maximal ankle dorsiflexion during stance phase of the gait (°)	4.5 (4.7)	6.0 (3.6)	*0.26*	7.8 (4.7)	8.2 (3.2)	*0.74*
Time at maximal ankle dorsiflexion during stance (% gait cycle)	24.3 (9.8)	22.1 (9.9)	*0.49*	29.0 (9.7)	33.9 (9.7)	*0.12*
Slope of the curve to estimate first rocker* (°/s)	**0.4 (0.6)**	**−0.2 (0.6)**	*0.02*	−0.8 (0.7)	−0.4 (0.7)	*0.07*
First/second ankle moment ratio	0.9 (0.3)	0.9 (0.2)	*0.99*	0.7 (0.2)	0.7 (0.2)	*0.80*
Maximal ankle power (W/kg)	3.2 (0.9)	3.3 (0.4)	*0.25*	**3.6 (1.0)**	**2.8 (0.7)**	*<0.05*

*Slope of the sagittal ankle kinematics during the first nine percent of the gait cycle. A negative value indicates a plantarflexion movement and so the presence of the first ankle rocker.

At the last CGA, compared with the ITW surgical group after treatment, significant differences were found between the two groups for the ankle passive dorsiflexion with extended and flexed knee with higher values for the ITW surgical group (Table 3). It was also observed a significant higher maximal ankle power for the ITW surgical group compared to ITW conservative group (3.6 W/kg (1.0) vs. 2.8 W/kg (0.7)). Kinematic and kinetic curves showed improvement after the both treatments with however a more consequent improvement for the group with conservative treatment.

### Surgical treatment in comparison with asymptomatic children

There were significant differences between post-operative surgical group and asymptomatic children for all measured parameters except for the maximal ankle power (Table 4). Ankle GVS was significantly superior in the surgical group, with a value of 6.7° (2.4) post-surgery and 3.8° (1.6) in asymptomatic children indicating an altered global pattern of gait in the surgical group. Maximal ankle dorsiflexion during stance was lower in the surgical group 8.0° (4.1) in post-surgery vs. 14.0° (2.0) in asymptomatic children, as well as time at maximal ankle dorsiflexion during stance meaning an earlier third rocker 30.8% (9.5) in post-surgery vs. 45.8% (5.7) in asymptomatic children. The slope of the sagittal ankle kinematics during the first nine percent of the gait cycle, representative of the first ankle rocker was less negative in the surgical group (−0.6°/s (0.7)) than in the asymptomatic children group (−1.1°/s (0.3)) and the first/second moment ratio was higher in the surgical group compared to the asymptomatic children group (0.7 (0.1) in post-surgery vs. 0.4 (0.2) in asymptomatic children). The Figs 2 and 3 showed that the kinematic and kinetic curves were improved but not normalized compared to the curves of the asymptomatic children.

**Table 4:** Mean and SD of kinematic/kinetic and physical examination outcome measurements in ITW surgical group after the surgery and in asymptomatic children.

Variables	Post-surgery n (legs) = 32	Asymptomatic children n (legs) = 42	*p-values*
Ankle passive dorsiflexion Extended knee (°)	10.3 (5.1)	No data	–
Ankle passive dorsiflexion Flexed knee (°)	16.9 (5.8)	No data	–
Ankle GVS (°)	**6.7 (2.4)**	**3.8 (1.6)**	*<0.05*
GPS modified (°)	**6.1 (1.2)**	**4.4 (0.9)**	*<0.05*
Maximal ankle dorsiflexion during stance phase of the gait (°)	**8.0 (4.1)**	**14.0 (2.0)**	*<0.05*
Time at maximal ankle dorsiflexion during stance (% gait cycle)	**30.8 (9.5)**	**45.8 (5.7)**	*<0.05*
Slope of the curve to estimate first rocker* (°/s)	**−0.6 (0.7)**	**−1.1 (0.3)**	*<0.05*
First/second ankle moment ratio	**0.7 (0.1)**	**0.4 (0.2)**	*<0.05*
Maximal ankle power (W/kg)	3.7 (1.1)	3.6 (0.8)	*0.75*

*Slope of the sagittal ankle kinematics during the first nine percent of the gait cycle. A negative value indicates a plantarflexion movement and so the presence of the first ankle rocker.

### Conservative treatment in comparison with asymptomatic children

Similar results for the conservative treatment group than for ITW surgical group were observed compared to asymptomatic children (Table 5) The maximal ankle dorsiflexion during stance was lower in the conservative group 8.7° (3.5) in post-treatment vs. 14.0° (2.0) in asymptomatic children as well as time at maximal ankle dorsiflexion during stance meaning an earlier third rocker 32.9% (9.9) in post-treatment vs. 45.8% (5.7) in asymptomatic children. However, contrary to surgical group, the maximal ankle power was significantly lower in the conservative group compared to the asymptomatic group (3.0 W/kg (0.8) in post-conservative treatment vs. 3.6 W/kg (0.8) in asymptomatic children). As the surgical group, the Figs 2 and 3 showed that the kinematic and kinetic curves were not normalized compared to the curves of the asymptomatic children.

**Table 5:** Mean and SD of kinematic/kinetic and physical examination outcome measurements in ITW after conservative treatment and in asymptomatic children.

Variables	Post-conservative treatment n (legs) = 26	Asymptomatic children n (legs) = 42	*p-values*
Ankle passive dorsiflexion Extended knee (°)	7.9 (6.9)	No data	–
Ankle passive dorsiflexion Flexed knee (°)	15.9 (7.1)	No data	–
Ankle GVS (°)	**6.3 (2.1)**	**3.8 (1.6)**	*<0.05*
GPS modified (°)	**6.0 (1.4)**	**4.4 (0.9)**	*<0.05*
Maximal ankle dorsiflexion during stance phase of the gait (°)	**8.7 (3.5)**	**14.0 (2.0)**	*<0.05*
Time at maximal ankle dorsiflexion during stance (% gait cycle)	**32.9 (9.9)**	**45.8 (5.7)**	*<0.05*
Slope of the curve to estimate first rocker* (°/s)	**−0.6 (0.7)**	**−1.1 (0.3)**	*<0.05*
First/second ankle moment ratio	**0.6 (0.2)**	**0.4 (0.2)**	*<0.05*
Maximal ankle power (W/kg)	**3.0 (0.8)**	**3.6 (0.8)**	*<0.05*

*Slope of the sagittal ankle kinematics during the first nine percent of the gait cycle. A negative value indicates a plantarflexion movement and so the presence of the first ankle rocker.

## Discussion

Our study highlights that, regardless of the treatment, the gait quality of the ITW patients improves over time but does not recover the gait of asymptomatic children.

Regarding more in details each group, it was observed the beneficial effects of surgery on gait deviations and on passive ankle flexion in ITW patients with a consequent improvement of the three criteria of severity established by Alvarez et al. thanks to surgery: the presence of first ankle rocker was improved, the third rocker was delayed and the first/second ankle moment ratio was reduced [18]. These results are in agreement with the literature [5]. Indeed, Hemo et al. found a significant increase of passive and active ankle dorsiflexion after surgical treatment [10]. Besides, improvement of ankle GVS was found by McMulkin and MacWilliams, showing a better ankle dorsiflexion pattern during gait [26]. Recently, Westeberry et al, analyzed the gait and the radiography of 26 patients with severe ITW, before and one year after surgery, consisting of Zone II or Zone III plantar flexor lengthening [9]. They observed postoperative improvements in ankle kinematics and kinetics in both zones lengthening without an increased risk of overlengthening, with no need for additional interventions and a decreased rate of recurrence. The same improvements have been observed for kinematic and kinetic curve’s patterns of this study, with a diminution of the variability at the last CGA showing a better repetability in their gait (Fig 2). As a matter of fact, overlengthening can be one of the main problems after lengthening of the Achilles tendon surgery with a consequence of muscle weakening, loss of the ankle plantar flexion-knee extension couple, inability to walk on toes, and in the worst case the development of crouch gait [5]. This complication is often observed for patients with persistent toe-walking associated with other disease as cerebral palsy, neuropathy, foot deformities or muscular dystrophy [27–29]. Thus, this problem was not observed in our cohort as for other authors publishing the same topic [9,30].

Regarding the evolution of ITW conservative group, significant improvements were also observed in this cohort for the majority of kinematic and kinetic outcomes and on the kinematic and kinetic curves (with also a diminution of the gait variability – Fig 2) after three years of follow-up. These observations were in link with the previous literature. Indeed, several authors found that ITW patients with conservative treatment have an improvement of their gait. Engström and Tedroff studied the natural history of ITW children from 5.5 to 10 years of age [4]. They found that about 80% of these children ceased toe-walking without treatment by the age of 10. However, ITW was assessed by a questionnaire, which is not as sensible as gait analysis. Davies et al. studied with gait analysis the long-term evolution of ITW with “active” conservative treatment (cast, botulin toxin injection) and “inactive” conservative treatment (stretching alone) [8]. They found in the two groups a diminution of the severity of ITW, and a cessation of toe walking in approximately half of the participants 9–18 years after treatment. Thus, natural history or stretching treatment alone of ITW seems to lead to a spontaneous decrease of the severity, and in some cases cessation of ITW. Recently, Bartoletta et al, found in their retrospective review of ITW patients without surgery, that patients with ankle foot orthoses were the only method associated with improvement [7]. Otherwise, Engström and Tedroff showed that ITW was not resolved spontaneously for children with severe contracture of the calf muscles [4]. Surgical treatments should, therefore, be reserved for children with severe ITW, complaints, no possibility to correct the gait pattern and contracture of the calf muscles.

This is in line with the present study showing that, for matched patients in terms of ankle passive dorsiflexion with the knee extended at baseline, ITW patients with surgery had still a more severe calf muscles contracture with a significant lower passive ankle movement with the knee flexed (e.g., gastrocnemius contracture), a lower first rocker or an absence of the first rocker, more pain in their ankles, calves or feet and a majority of them were not able to walk in a typical heel-foot progression compared to ITW patients with conservative treatment (Table 3). The kinematic and kinetic curves showed also at the last CGA an improvement for the two groups in term of gait pattern but patients with conservative treatment have less improvement than patients with surgery (Fig 3). However, at the last CGA, this group had no more pain with a higher ankle passive dorsiflexion and higher push-off at the ankle joint. The surgery for patients with severe equinus appears to reduce strain and compensatory stress on the lower limb that may have previously contributed to pain during walking. Consequently, the surgical treatment has a positive effect on gait quality, function, pain without loss of push-off. Only few studies compared conservative and surgical treatments. Stricker and Angulo compared ankle passive dorsiflexion and parental satisfaction after observation, conservative treatment or surgical triceps surae lengthening [13]. They found better passive ankle dorsiflexion and parental satisfaction in the surgical group compared to the other care. However, surgery was used only for the worst cases with fixed equinus contractures and no instrumented gait analysis was performed. In a systematic review of the literature, Van Bemmel et al. concluded that the results of existing studies were not able to establish the superiority of surgery or conservative treatments [2].

Nevertheless, Westeberry et al., despite a good evolution of their outcomes, did not observe a return to normal gait one year after surgery, as is the case in this current study showing significant differences for all the outcomes analyzed except maximal ankle power between surgically treated ITW patients and asymptomatic children [9]. The kinematic and kinetics curves illustrated also that at the last CGA, the gait patterns were not normalized (Figs 2 and 3). Thus, Achilles tendon lengthening does not enable a return to a normal gait of ITW patients but there is no reduction in maximal ankle power. Stott et al. found similar results: they performed gait analysis on seven skeletally mature patients who had surgery of lengthening of Achilles tendon or gastrocnemius-soleus for ITW, with a mean follow-up of 10.5 years: most children still had persistent deviations in ankle kinematics and kinetics [12]. However, two of their seven subjects had a maximal ankle power just below the normal average, contrary to the results of the current study.

This study has several limitations. The size of the two samples was small with only 16 patients in the surgical group and 13 in the conservative group. However, comparing with other studies including ITW patients with CGA the sample size is similar. Another limitation is the retrospective design and the presence of a selection bias as the surgical group presented slightly more severe ITW patients than the conservative group. This bias reflects also the reason of the surgery compared to the conservative group. A randomised study with more participants would allow a better assessment of the benefits of surgery compared to conservative treatment.. Finally, the time of the follow-up period is short and should be longer with the need to also include an adolescent population to have an idea of the impact of morphological changes on functional consequences.

In conclusion, this study shows that gait outcomes improved after surgery in ITW but they did not recover a normal gait. As gait is also improved after conservative treatment, surgical treatments should, therefore, be reserved for children with severe ITW, complaints about pain, and contracture of the calf muscles. Further randomized controlled studies are needed with a larger sample and a longer follow-up.
